# Can Damage to the Rat Lung Induced by Prolonged Normobaric Hypoxia and Norepinephrine Be Reversed by Normoxic Recovery?

**DOI:** 10.3390/cimb47110931

**Published:** 2025-11-08

**Authors:** Sarah Daunheimer, Charly Bambor, Coralie Raffort, Julia Koedel, Aida Salameh, Beate Rassler

**Affiliations:** 1 Carl-Ludwig-Institute of Physiology, Medical Faculty, Leipzig University, 04103 Leipzig, Germany; sarah.daunheimer@gmx.de (S.D.); charly.bambor@gmx.net (C.B.); 2 Department of Pediatric Cardiology, Heart Centre, Leipzig University, 04289 Leipzig, Germany; coralie.raffort@uni-leipzig.de (C.R.); aida.salameh@medizin.uni-leipzig.de (A.S.); 3 Institute of Pathology, Medical Faculty, Leipzig University, 04103 Leipzig, Germany; julia.koedel@medizin.uni-leipzig.de

**Keywords:** hypoxia, norepinephrine, pulmonary edema, oxidative stress, inflammation, capillary wall stress, capillary stress failure

## Abstract

Exposure to hypoxia may cause lung injury characterized by hydrostatic pulmonary edema (PE), inflammation and oxidative stress. Norepinephrine (NE) infusion can also induce lung injury with similar pathogenetic characteristics. The main questions of this study were (i) whether NE infusion aggravates hypoxia-induced pulmonary injury; (ii) whether inflammation and oxidative stress deteriorate the hypoxic PE; and (iii) whether PE and inflammation recede after three days of normoxic recovery. Ninety-eight female rats were exposed for 72 h to normoxia or normobaric hypoxia and received infusions with NaCl or NE. Some of these animals were transferred to a three-day normoxic recovery period thereafter. We performed histological and immunohistochemical analyses of the lung, determined protein concentrations in pleural fluid (PF) and bronchoalveolar lavage fluid (BALF), and evaluated hemodynamic parameters. While inflammation and oxidative stress receded after 3 days of normoxic recovery, PE did not resolve. Increased protein concentrations in PF and BALF indicated that capillary stress failure increased even further during the normoxic recovery phase, particularly in animals that had previously received an NE infusion. These results highlight the fact that inflammation does not play a causal role in the development of hypoxic PE.

## 1. Introduction

Exposure to a hypoxic environment such as at high altitudes can exert detrimental effects on the lungs. The best-known lung damage caused by hypoxia is pulmonary edema (PE), e.g., high-altitude pulmonary edema (HAPE). Hypoxia-induced PE including HAPE is often accompanied by an inflammatory reaction in the lung [1,2,3,4], which, however, is not considered the cause of the PE [5]. With regard to its pathogenesis, hypoxic pulmonary edema is classified as non-cardiogenic hydrostatic edema. A markedly increased pulmonary capillary pressure resulting from an uneven hypoxic pulmonary vasoconstriction is considered to be the main cause of PE. An important component of hypoxic pulmonary vasoconstriction is a reduced bioavailability of nitric oxide (NO) [6,7,8]. In pulmonary regions with strong vasoconstriction, perfusion is low while regions with weak vasoconstriction are overperfused. In these areas, capillary pressure increases and causes an enhanced filtration of fluid from the vessels into the interstitium of the lung [9]. In severe cases of PE, the drainage pathways of the lung into the lymph vessels and the pleural space [10,11,12] are not sufficient. As the mechanisms preventing edema formation are limited, flooding of the alveoli may occur [13]. For mountaineers at high altitudes, a rapid descent to lower altitudes and hence, the attenuation of hypoxia, is the most effective therapeutic measure. If descent is impossible, improvement of oxygenation can be achieved by supplemental oxygen or a hyperbaric bag. If all these possibilities are not available, drug treatment with nifedipine or sildenafil is recommended [14].

Although hypoxia-induced PE is mainly a hydrostatic edema, it is commonly accompanied by inflammation. Both in patients at high altitudes with HAPE and in rats under conditions of normobaric hypoxia with 10% O_2_, significant increases in the number of inflammatory cells such as alveolar macrophages and lymphocytes as well as in the expression of tumor necrosis factor (TNF)α and other inflammatory mediators were shown in lung tissue and in bronchoalveolar lavage (BAL) fluid [1,2]. Alveolar hypoxia induces the accumulation and activation of leukocytes, in particular macrophages, lymphocytes and monocytes in the lung [15]. These cells initiate the inflammatory response, which is an important mechanism of defense in the lower respiratory tract. Macrophages, which are the main source of cytokines and other inflammatory mediators such as TNFα, reside in airways, the alveoli and in the lung interstitium, that means, in regions that are most directly exposed to inhaled pathogens [16]. In previous studies, we also demonstrated significantly elevated levels of TNFα in the bronchial and peribronchial regions in rats exposed to normobaric hypoxia over 24–72 h, indicating that hypoxic PE was accompanied by inflammation [3,4]. However, in the early stage of HAPE, inflammatory cells and cytokines in BAL fluid were in a normal, that means, a non-inflammatory, range. An increase in inflammation markers was only observed in the late stages of hypoxia [17]. Therefore, inflammation is considered to be a consequence rather than a cause of PE [5].

In addition to PE and inflammation, hypoxia-induced lung injury is associated with oxidative stress resulting from an excess of reactive oxygen species (ROS), or more comprehensive, reactive oxygen nitrogen species (RONS) with the most important representatives being superoxide, hydrogen peroxide, nitric oxide and peroxynitrite. Hypoxia can lead to increased production of RONS through several mechanisms, mainly mitochondrial dysfunction and the activation of various pro-oxidant enzymes [18]. Reduced oxygen availability leads to a limited electron flow rate in the mitochondrial respiratory chain, resulting in increased electron leakage. This promotes the reduction of molecular oxygen to superoxide, especially during partial reoxygenation [19]. In addition, hypoxia-inducible factors (HIFs), the main regulators of the hypoxia response, also contribute to the upregulation of genes encoding RONS-generating enzymes such as NADPH oxidase, which causes more electrons to be transferred from NADPH to oxygen [20]. This results in an increased formation of superoxide and hydrogen peroxide [21]. During reoxygenation after a hypoxic phase, xanthine oxidase activity may also increase, contributing to excessive RONS production. This process, also known as reperfusion stress, exacerbates oxidative damage and can activate inflammatory signaling pathways [22]. Although RONS can be involved in cellular adaptation to stress and hypoxia, the overproduction of RONS can lead to damage to cells, proteins and DNA. Peroxynitrite, which is formed by combination of NO and superoxide, is a highly tissue-toxic RONS that can impair the normal function of the vascular endothelium and increase vascular permeability [23]. Peroxynitrite can be detected by its degradation product nitrotyrosine, which serves as an important marker of oxidative/nitrosative stress [24]. Furthermore, RONS can activate various signaling pathways involved in the production of proinflammatory cytokines and thus promote inflammation in the lung. In turn, inflammatory cells release RONS, further increasing oxidative stress. This results in a vicious cycle that may progressively deteriorate hypoxic lung damage [18,25].

Hypoxia is associated with activation of the sympathetic nervous system (SNS). Strong sympathetic activation or increased catecholamine levels in the blood can also induce PE. Animal studies showed that even under normoxic conditions, the administration of epinephrine or norepinephrine (NE) led to the formation of partly severe PE accompanied by inflammation [26,27,28]. Massive activation of the SNS, as may arise from a severe head or spinal cord injury [29,30], can induce an acute severe PE, which is termed neurogenic PE. PE is also a serious complication of pheochromocytoma, a catecholamine-producing tumor of the adrenal medulla or other paraganglia [31,32]. One of the main pathogenic mechanisms of catecholamine-induced PE is a strong vasoconstriction that also includes pulmonary veins [30,33]. Similarly to hypoxia, this leads to increased pulmonary capillary pressure. In addition, adrenergic agonists, in particular α-adrenergic stimulators, also exert proinflammatory effects, which may aggravate hypoxic lung injury. However, previous studies on rats under hypoxic conditions receiving adrenergic blockers indicated that the contribution of hypoxia-induced activation of the SNS to the development of hypoxic PE is rather small [4,34]. Travelers to high altitudes are advised to spend at least the first day at rest and to avoid physical exertion. Hence, one important aim of the present study was to investigate whether stronger SNS activation might aggravate complications of hypoxia such as hypoxic lung injury. As a model of strong sympathetic activation, we applied infusion with NE. Secondly, we studied whether inflammation and oxidative stress would deteriorate hypoxic PE. Finally, we examined whether pulmonary edema and inflammation would recede after a three-day recovery period under normoxic conditions and infusion with 0.9% NaCl solution.

## 2. Materials and Methods

### 2.1. Animal Model

All experiments were performed on 98 female Sprague–Dawley rats supplied by Charles River (Sulzfeld, Germany) aged about 10–12 weeks. Their body weight was 242.8 ± 1.9 g at the beginning of this study. All animal protocols were approved by the Federal State Agency (Landesdirektion Sachsen, protocol number TVV 46/18). The experiments were conducted in accordance with the Guide for the Care and Use of Laboratory Animals published by the National Institutes of Health and with the “European Convention for the Protection of Vertebrate Animals used for Experimental and other Scientific Purposes” (Council of Europe No 123, Strasbourg 1985).

### 2.2. Study Protocol

Animals were subdivided into two cohorts for exposure to normoxia (N, *n* = 50) or normobaric hypoxia (H, *n* = 48) for 72 h. The equipment for exposure to hypoxia was as previously described [35,36]. In brief, the animals were placed into a hypoxic chamber with 10% oxygen in nitrogen, while normoxic animals remained under room air conditions. Additionally, all animals received an intravenous infusion over the total experimental time with external automatic syringe pumps (Infors AG, Basel, Switzerland) at a rate of 0.1 mL h^−1^ via an infusion catheter (Vygon, Aachen, Germany) that was placed in the animal’s left jugular vein. The catheter was tunneled under the skin to the back of the animal and fixated with some stitches to the skin. Within both the normoxic and hypoxic cohorts, one group was infused with 0.9% sodium chloride (NaCl) solution; another group was administered NE (0.1 mg kg^−1^ h^−1^). The experiment ended after 72 h for some of the animals of those 4 groups. These four subgroups were referred to as N-NaCl (*n* = 14), H-NaCl (*n* = 18), N-NE (*n* = 14) and H-NE (*n* = 10). The remaining animals were placed for another 72 h under normoxic conditions with NaCl infusion as recovery groups, labeled as N-NaCl+R (*n* = 8), H-NaCl+R (*n* = 8), N-NE+R (*n* = 14) and H-NE+R (*n* = 12).

The infusion catheter was inserted into the left jugular vein under anesthesia with 2% isofluran. After catheter insertion, the animals woke up and moved freely with access to tap water and a rat chow diet (Altromin C100, Altromin GmbH, Lage, Germany). Exposure to a hypoxic environment started immediately after catheter insertion. The oxygen concentration in the chamber as well as a sufficient availability of food and drinking water were checked regularly.

### 2.3. Hemodynamic Measurements and Sampling of Materials

About 50 min before the end of the experiment, the animals were anesthetized with an intraperitoneal injection of thiopental (Trapanal^®^ 80 mg kg^−1^). Thereafter, a trache-otomy was performed and a polyethylene cannula was placed in the trachea. As previously described (see our previous publication [35]), the right ventricle (RV) was catheterized with a Millar^®^ (Millar Instruments, Houston, TX, USA) ultraminiature catheter pressure transducer and the left ventricle (LV) was catheterized with a pressure–volume catheter (Millar Instruments, Houston, TX, USA). Data acquisition and analysis were performed with Power Lab and Lab Chart Software from ADInstruments (version 8.1.9, ADInstruments Europe/UK, Oxford, UK) and a modified LabChart Software (version 1.0) from the ADInstruments sales department (FMI Föhr Medical Instruments GmbH, Seeheim, Germany). We measured left and right ventricular systolic peak pressures (LVSP, RVSP), left ventricular end-diastolic pressure (LV edP), stroke volume (SV) and heart rate (HR). After withdrawing the LV catheter tip into the aorta, the mean aortic pressure (MAP) was measured. The cardiac index (CI, body mass-related cardiac output) was determined by thermodilution using a thermosensitive 1.5F microprobe and a Cardiomax II computer (Columbus Instruments, Columbus, OH, USA). Total peripheral resistance (TPR) was calculated by dividing MAP by CI.

Animals of the H-NaCl and H-NE groups remained in hypoxia until the completion of hemodynamic measurements. After termination of the hemodynamic measurements, the abdominal cavity was opened by midline incision. Animals were sacrificed by drawing blood from the abdominal aorta. The blood was centrifuged for 10 min at 2100 rpm. Then, we opened the thoracic wall, collected pleural fluid (PF) and measured the PF volume. The right bronchus was ligated, and a bronchoalveolar lavage (BAL) of the left lung was performed two times consecutively with 3 mL 0.9% NaCl each. The fluid was instilled via the tracheal cannula into the left lung and withdrawn immediately. While the left lung was discarded, tissue samples from the intact right lung were fixated in formalin for histological and immunohistochemical analyses. Serum, PF and BAL fluid (BALF) were frozen and stored at −80 °C for analysis of protein content.

### 2.4. Lung Histology

The formalin-fixated tissue samples of the right lung were embedded in paraffin, sliced, and stained with hematoxylin–eosin (H&E). Histological assessments were performed by two independent investigators (S.D. and J.K.) who were blinded to the treatment group. They evaluated PE and blood congestion in the total section area of the upper and lower lung lobes. A detailed quantification of PE was performed as described elsewhere [4]. In brief, PE severity (expressed as PE score ranging from 0 to 3) was assessed by evaluating the width of the alveolar septa and the definition of alveolar spaces (for more details see [4]. The PE scores ranged from 0 to 3 and were defined as follows:

- 0, no PE.
- 1, mild PE: alveolar septa slightly thickened, alveolar space well defined.
- 2, moderate PE: thickness of alveolar septa about twice the normal width, alveolar space narrowed but still defined.
- 3, severe PE: alveolar spaces hardly determinable and/or alveolar edema.

The PE index (PEI) was calculated by cumulating the products of the PE score and the proportionate area of each part of the histological preparation, providing one value per animal. The congestion index was determined in an analogous way with the following:

- 0, no congestion.
- 1, mild congestion, in the interstitium only.
- 2, moderate congestion, alveolar walls affected as well.
- 3, severe congestion, hemorrhage, erythrocytes in the alveolar spaces.

### 2.5. Immunohistochemistry

Immunohistochemical analysis was applied as described previously [4] to determine the expressions of TNFα as a potent proinflammatory cytokine, and of nitrotyrosine (NT) as a marker of oxidative stress in the lungs. In brief, 2 μm thick sections of the right lung were dewaxed, rehydrated, cooked in 0.01 M citrate buffer (pH = 6) and then blocked with bovine serum albumin (BSA) to saturate unspecific bindings. The specimens were treated with the primary antibodies overnight at 4 °C. For the determination of TNFα, we used rabbit monoclonal anti-TNFα primary antibodies (catalog number SAB4502982, original concentration 1 mg/mL, diluted to 1:100, Sigma-Aldrich, Taufkirchen, Germany), and for the detection of NT, mouse monoclonal anti-nitrotyrosine primary antibodies (catalog number MAB5404, original concentration 1 mg/mL, diluted to 1:100, Merck-Millipore, Darmstadt, Germany) were employed. The next day, the specimens were washed and the appropriate horseradish peroxidase-labeled secondary antibody was applied for 1 h (TNFα) or 2 h (NT) at room temperature. We used a goat anti-rabbit antibody (catalog number A0545, 1:200) for TNFα (Sigma-Aldrich, Taufkirchen, Germany), and a goat anti-mouse antibody (catalog number 12-349, original concentration 1 mg/mL, diluted to 1:200) for NT (Merck-Millipore, Darmstadt, Germany). After another wash cycle, visualization of positive cells was performed with AEC red chromogen (Enzo, Lörrach, Germany). Cell nuclei were counterstained with hemalum.

Microscopic examination and photography were performed using the Axioimager M1 microscope from Zeiss (Carl Zeiss, Jena, Germany) together with an AxioCam MRc 5 camera and Zen Blue 3.1 software (Carl Zeiss, Jena, Germany). For the detection of TNFα, which is mainly located in the bronchial and peribronchial regions, at least 8 photographs per animal were taken from these regions at 200× magnification (20× through the objective lens, 10× through the eyepiece). For NT detection, we took at least 50 photographs per animal at 50× magnification (5× through the objective lens, 10× through the eyepiece) to evaluate the entire sections of the upper and lower lung lobes. The program ImageJ (version 1.54g) [37] was used for measurements of the TNFα- or NT-positive areas (in µm^2^) appearing in red in the pictures. The expression of TNFα is given as the TNFα-positive area related to the bronchial surface area of the specimen (in percent). The expression of NT is given as the NT-positive area related to the total lung area of the specimen (in percent).

### 2.6. Protein Concentration in Serum, BALF and PF

Total protein concentration in the fluids was determined using the BCA protein assay from Pierce (catalog number 23227, Thermo Fisher Scientific, Dreieich, Germany), according to the manufacturer’s instructions. For the analysis of BALF, the undiluted supernatant of the first lavage was used. Pleural fluid (PF) and serum (S) were diluted with distilled water at a ratio of 1:200. A standard curve was generated with bovine serum albumin (25 µg/mL to 2000 µg/mL) and measured together with the unknown samples at 562 nm. We used the spectrophotometer Synergy HTX form BioTek (now Agilent, Waldbronn, Germany) for a twofold determination of the protein concentrations [P]_S_, [P]_PF_, and [P]_BALF_ (given in g/L). The concentration ratios [P]_PF_/[P]_S_ and [P]_BALF_/[P]_S_ were calculated for each individual animal and are given in %.

### 2.7. Statistical Analysis

All data are given as mean ± SEM. Statistical analyses were carried out with the software package SigmaPlot Version 16.0 (Systat Software GmbH, Erkrath, Germany) for Windows. All groups were statistically compared using analysis of variance (ANOVA) procedures. At first, a Shapiro–Wilk test of normality was conducted. If the data were normally distributed, we performed a one-way ANOVA with post hoc tests according to Fisher’s method of least significant differences (LSD). If the data were not normally distributed, a Kruskal–Wallis ANOVA on ranks with a post hoc test according to Dunn’s method was applied. *p* values < 0.05 were considered significant.

## 3. Results

### 3.1. Pulmonary Injury

#### 3.1.1. Pulmonary Edema

**Figure 1:**
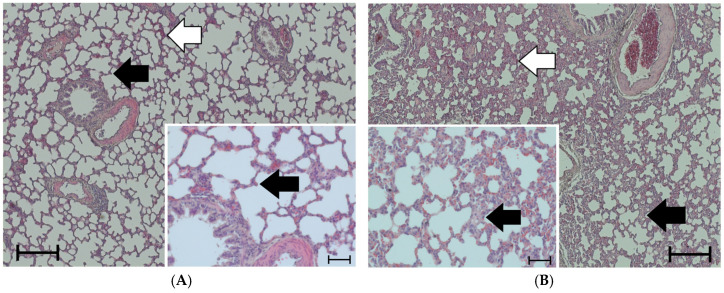

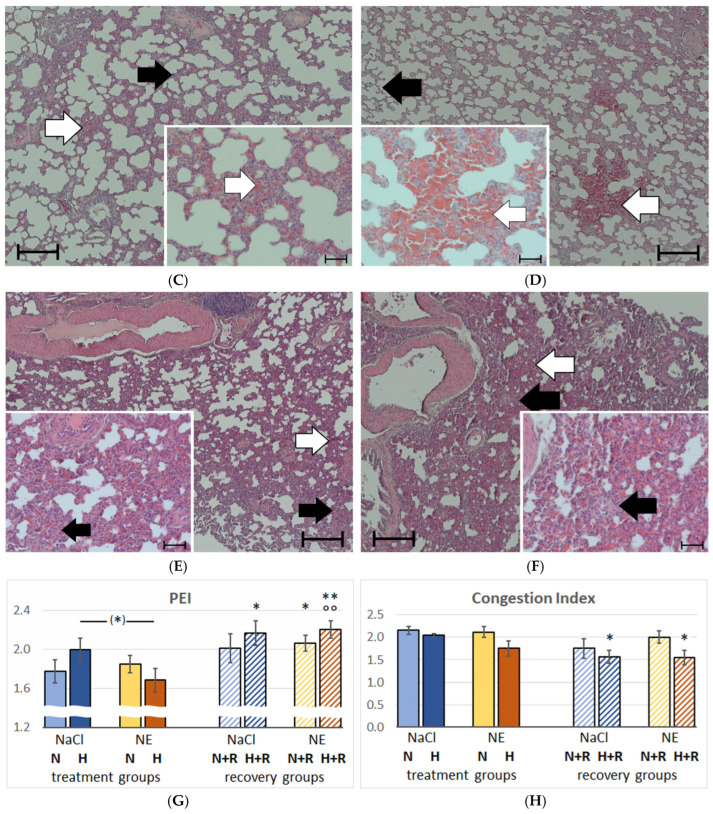
Pulmonary edema and pulmonary blood congestion. The panels (**A**–**F**) show representative histological images (50× magnification) with insets (200× magnification) to demonstrate relevant details. The scale bars for 50× magnification correspond to 200 μm. The scale bars for the insets (200× magnification) correspond to 50 µm. Pulmonary edema is indicated by black arrows; blood congestion is indicated by white arrows. (**A**): N-NaCl, mild PE, mild congestion; the inset shows an area with no or mild PE, as characterized by thin alveolar septa. (**B**): H-NaCl, moderate PE, mild-to-moderate congestion; the inset shows that the alveolar septa are thickened. (**C**): N-NE, moderate PE, moderate congestion; the inset shows an area with moderate blood congestion, as indicated by numerous erythrocytes in the alveolar septa. (**D**): H-NE, mild PE, moderate congestion; the inset shows an area with moderate blood congestion; erythrocytes have entered an alveolus, which occurred in rare cases. (**E**): H-NaCl+R, severe PE, moderate congestion; the inset shows severely thickened alveolar walls, with constricted alveolar spaces. (**F**): H-NE+R, severe PE, moderate congestion; the inset shows a similar picture as in panel (**E**), with the alveolar spaces hardly determinable. (**G**): Pulmonary edema index (PEI); (**H**): congestion index, both expressed in arbitrary units. Data are given as means ± SEM. Significant differences: vs. N-NaCl: * *p* < 0.05, ** *p* < 0.01; vs. corresponding treatment group: °° *p* < 0.01. The symbol (*) indicates an almost significant difference between the marked groups with *p* = 0.053.

Lung histology revealed the existence of edema in the lungs of both normoxic and hypoxic rats (Figure 1A–F). Under hypoxic conditions, the type of infusion had different effects on the PE’s severity. While the PE mildly deteriorated with NaCl infusion, NE tended to reduce the PEI compared to the H-NaCl group (*p* = 0.053). However, contrary to what we had expected, the edema did not recede during the recovery period, but instead became even more severe, particularly in those groups that were previously exposed to hypoxia (Figure 1E–G). In general, the PE was confined to the interstitium, with the alveoli remaining free of fluid. An alveolar edema was observed in only one animal.

In addition, the histology also showed signs of blood congestion and inflammation (Figure 1A–F). Blood congestion appeared as accumulations of erythrocytes in the wall of the alveoli. In rare cases, we found erythrocytes in the alveolar lumen (for an example see Figure 1D). Of note is that the degree of blood congestion was not positively correlated with the degree of PE. The congestion index was higher in the N-NaCl group and decreased in the H-NaCl+R and H-NE+R groups (Figure 1H).

#### 3.1.2. Pulmonary Inflammation

**Figure 2:**
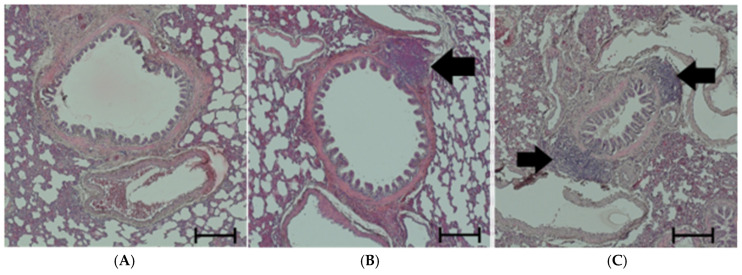
Lymphocyte accumulation in the lung, predominantly in bronchial walls. All images have 50× magnification. The scale bars correspond to 200 μm. Representative histological images from (**A**): N-NaCl: no prominent lymphocyte accumulations; (**B**): H-NaCl; (**C**): H-NE; lymphocyte accumulations can be found in the bronchial walls as marked by black arrows.

**Figure 3:**
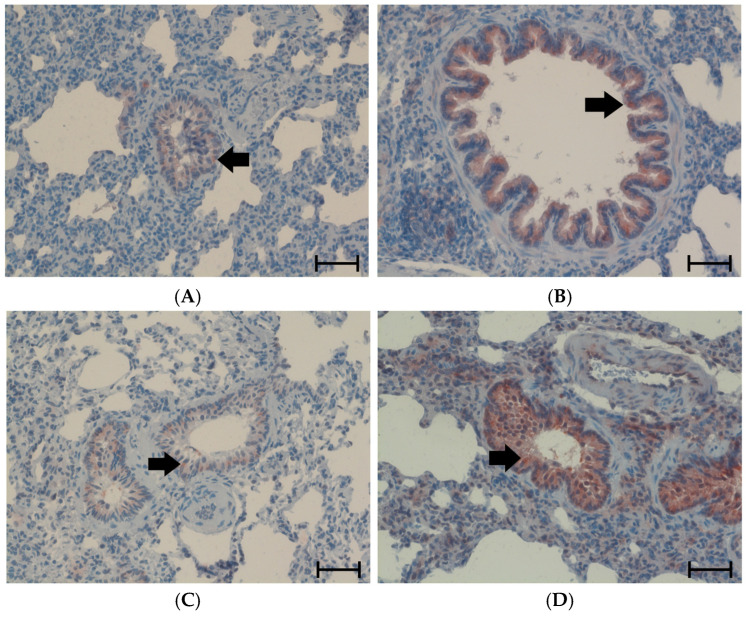

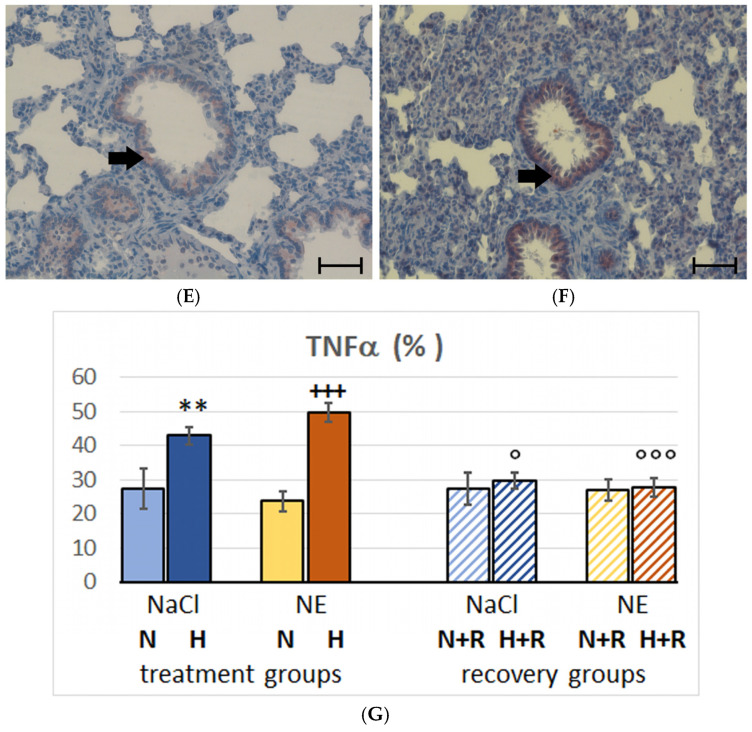
Tumor necrosis factor alpha (TNFα) in the lung. All images have 200× magnification. The scale bars correspond to 50 μm. Representative immunohistological images from (**A**): N-NaCl; (**B**): H-NaCl; (**C**): N-NE; (**D**): H-NE; (**E**): H-NaCl+R; (**F**): H-NE+R. TNFα is mainly located in the bronchial walls as marked by black arrows; TNFα-positive cells appear red. (**G**): abundance of TNFα in the groups expressed as positive area related to the bronchial surface area of the specimen [%]. Data are given as means ± SEM. Significant differences: vs. N-NaCl: ** *p* < 0.01; vs. N-NE: +++ *p* < 0.001; vs. corresponding treatment group: ° *p* < 0.05, °°° *p* < 0.001.

The PE was accompanied by inflammation, which was first noticed in the histological H&E preparations as accumulations of lymphocytes in the bronchial walls and their vicinity (Figure 2). TNFα is one of the most important proinflammatory cytokines in the lung. It has been detected immunohistochemically, predominantly in the walls of large, medium-sized and small bronchi. Its expression increased significantly under hypoxic conditions with NaCl infusion, and even more with NE infusion compared to the related normoxic groups. After three days of normoxic recovery, the TNFα levels completely regressed to the control level (Figure 3).

#### 3.1.3. Oxidative Stress in the Lung

**Figure 4:**
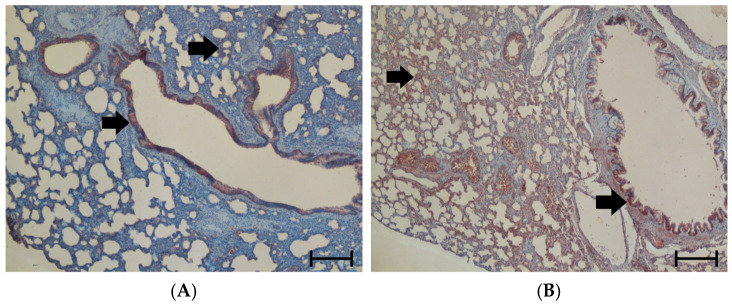

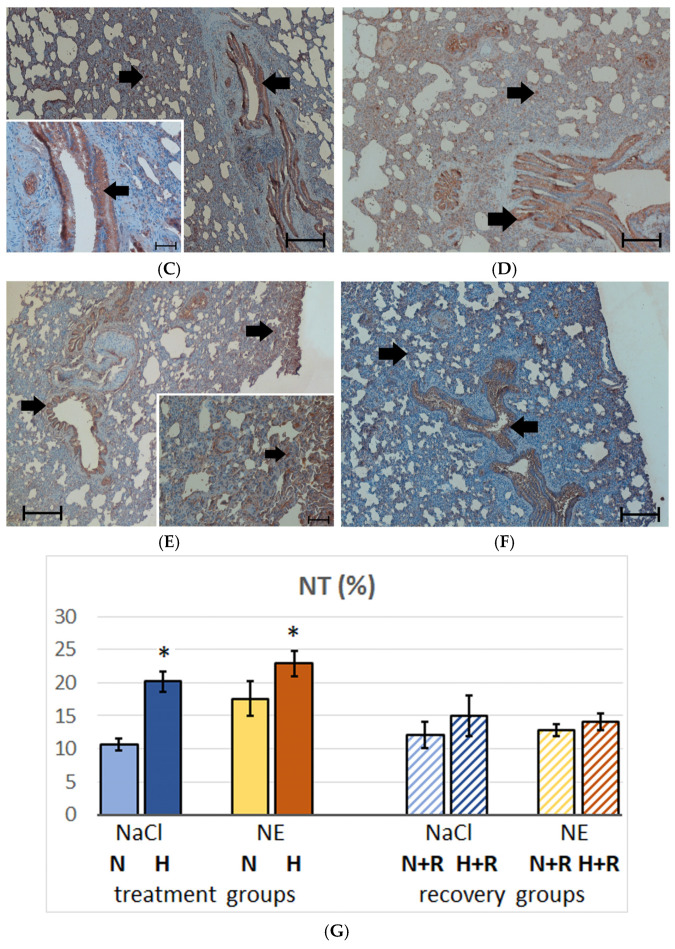
Nitrotyrosine (NT) in the lung. The panels (**A**–**F**) show representative histological images (50× magnification). Panels (**C**,**E**) contain insets (200× magnification) to show NT-positive areas in more detail. The scale bars for 50× magnification correspond to 200 μm. The scale bars for the insets (200× magnification) correspond to 50 µm. NT is located in both bronchial and peribronchial regions and in the entire interstitium as marked by black arrows; NT-positive cells appear red. (**A**): N-NaCl. (**B**): H-NaCl. (**C**): N-NE; the inset shows an NT-positive area in the bronchial wall. (**D**): H-NE. (**E**): H-NaCl+R; the inset shows an NT-positive area in the interstitium. (**F**): H-NE+R. (**G**): abundance of NT in the groups expressed as a positive area related to the total lung area of the specimen [%]. Data are given as means ± SEM. Significant differences vs. N-NaCl: * *p* < 0.05.

NT is a degradation product of the RONS peroxynitrite and, thus, serves as marker of oxidative stress. NT-positive cells have been found in bronchial and peribronchial cells, but they were also distributed throughout the entire interstitium (see arrows in Figure 4). Similarly to TNFα, its expression increased significantly under hypoxic conditions (H-NaCl and H-NE) and returned to the control level after three days of normoxic recovery (Figure 4).

### 3.2. Pleural Fluid and BALF

**Figure 5:**
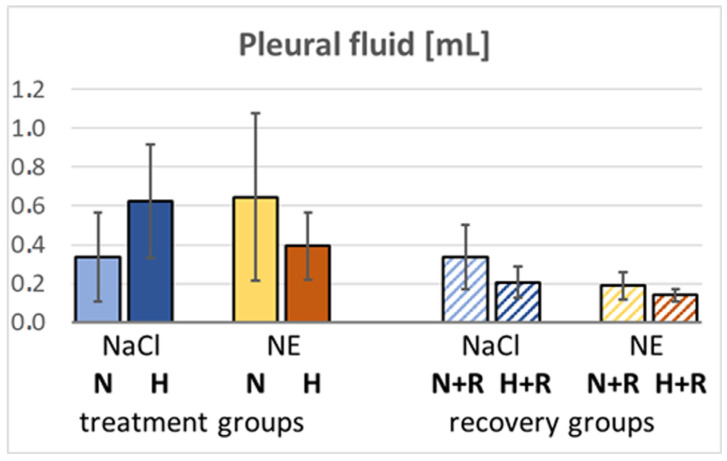
Pleural fluid volume [mL]. Data are given as means ± SEM.

During the formation of PE, fluid filtration into the pleural space can serve as a drainage route to reduce fluid overload in the interstitium. Animals exposed to hypoxia or infused with NE under normoxic conditions presented higher amounts of PF than normoxic ones or those with hypoxia and NE infusion. Of note, after 3 days of normoxic recovery, animals showed an increase in PE but markedly lower levels of PF (Figure 5).

**Figure 6:**
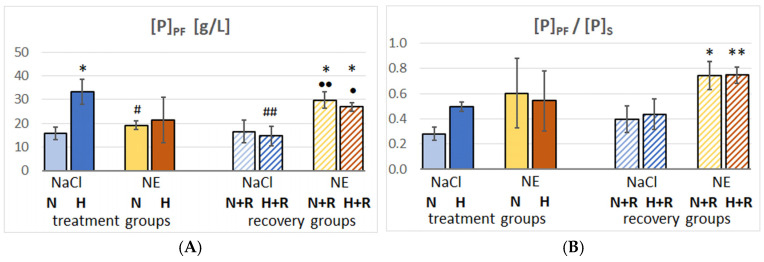

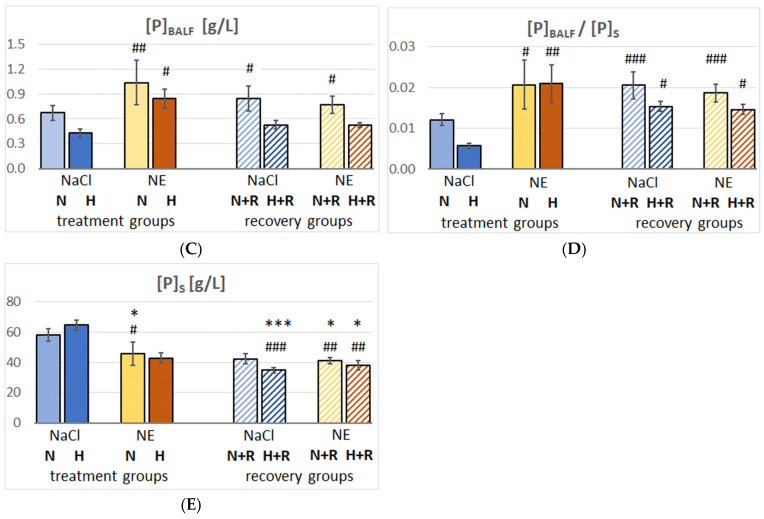
Protein concentrations [P] and protein concentration ratios. (**A**): [P] in pleural fluid (PF), in g/L; (**B**): protein concentration ratio between PF and serum (S); (**C**): [P] in bronchoalveolar lavage fluid (BALF), in g/L; (**D**): protein concentration ratio between BALF and serum (S); (**E**): [P] in serum (S), in g/L. Data are given as mean ± SEM. Significant differences: vs. N-NaCl: * *p* < 0.05, ** *p* < 0.01, *** *p* < 0.001; vs. H-NaCl: # *p* < 0.05, ## *p* < 0.01, ### *p* < 0.001; vs. H-NaCl+R: • *p* < 0.05, •• *p* < 0.01.

The total protein concentration ([P]) in PF, BALF and serum (S) as well as their ratios [P]_PF_/[P]_S_ and [P]_BALF_/[P]_S_ (Figure 6) provide further insights into PE. In the N-NaCl group, [P]_PF_ was in a normal range (<20 g/L), but hypoxia increased significantly to >30 g/L (H-NaCl) suggesting pleural exudation. The concentration ratio [P]_PF_/[P]_S_ of 0.5 confirmed this suggestion. With NE infusion, [P]_PF_ increased only slightly in normoxia (N-NE) and hypoxia (H-NE), but the ratio [P]_PF_/[P]_S_ was also above 0.5 (Figure 6A,B). In contrast, [P]_BALF_ and [P]_BALF_/[P]_S_ were significantly elevated in the NE groups, but not in the H-NaCl group (Figure 6C,D). Total serum protein concentration ([P]_S_) decreased in the NE-treated groups (Figure 6E).

After 72 h of normoxic recovery with NaCl infusion, [P]_PF_ returned to the control level in the group previously treated with H-NaCl, but increased in the animals that had previously been treated with NE. This was also reflected in the ratio [P]_PF_/[P]_S_. On the contrary, [P]_BALF_ increased in the NaCl groups after recovery while it slightly decreased in the groups with previous NE treatment, but in all groups it was higher than in the H-NaCl group. This is even more clearly reflected in the ratio [P]_BALF_/[P]_S_. [P]_S_ was low in all recovery groups.

### 3.3. Hemodynamic Results

**Figure 7:**
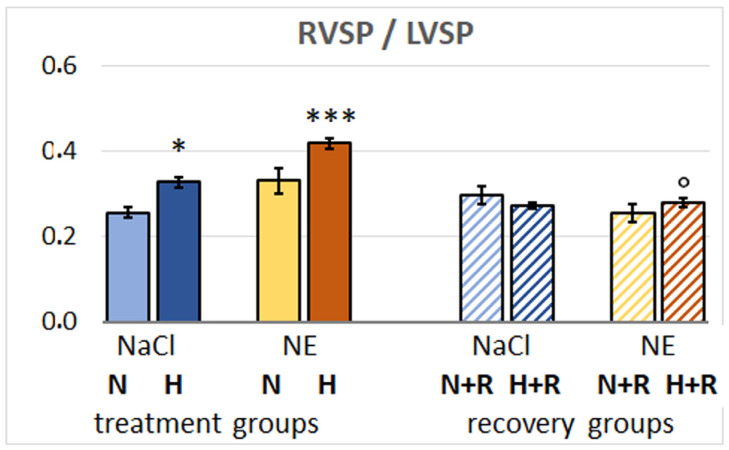
Ratio of right to left ventricular systolic pressure (RVSP/LVSP) indicating imbalance between systolic functions of RV and LV. Significant differences: vs. N-NaCl: * *p* < 0.05, *** *p* < 0.001; vs. corresponding treatment group: ° *p* < 0.05.

The hemodynamic results are presented in Table 1. Hypoxia induced a significant decrease in LVSP. NE infusion did not prevent this LVSP decrease. In contrast, the combination of NE infusion and hypoxia further deteriorated the LVSP depression significantly compared to the effect of hypoxia alone. In contrast to the LV, RVSP slightly increased under hypoxia and even more under hypoxia plus NE infusion, resulting in a significant mismatch between LV and RV pressures, presented as ratio of RVSP to LVSP (Figure 7). MAP and SV showed similar changes under hypoxia and/or NE infusion with the lowest values also observed in the H-NE group. HR decreased under hypoxia, but increased with NE infusion, so that the HR in the H-NE group was approximately at the level of the normoxic control. However, this effect was not sufficient to fully compensate for the reduction in LVSP and SV. The CI decreased in the H-NaCl group and even more in the NE groups with the lowest value in the H-NE group. Of note, LV edP increased slightly in the H-NaCl group but showed a significant increase in the H-NE group to about 150% of the normoxic control group. To summarize, animals in the H-NE group presented with the most severely impaired LV function, but also with the highest RVSP and thus the most pronounced imbalance between LV and RV pressures (see Figure 7). The TPR decreased slightly under hypoxia alone and increased with NE infusion, but these changes were not significant.

After three days of normoxic recovery with NaCl infusion, LVSP and MAP completely recovered; thus, eliminating the imbalance between the left and right ventricles. Even more so, in the N-NE+R group, these parameters even slightly exceeded the baseline values observed in the 72 h normoxic control animals. However, in the N-NaCl+R group, both LVSP and MAP were significantly lower than after 72 h of N-NaCl treatment. RVSP did not significantly change in the recovery groups. SV completely recovered in the H-NaCl+R group but was only partially restored in the N-NE+R group. However, in the H-NE+R group, SV significantly exceeded the normoxic control values. HR and CI also reverted in the H-NaCl+R group to normoxic control levels but did not fully recover in the groups previously infused with NE. LV edP returned in all recovery groups to the level of the normoxic control group. TPR was higher in all recovery groups than in the N-NaCl group, with the exception of the N-NaCl+R group.

## 4. Discussion

The main result of the present study was that both the hypoxia-induced depression of LV function and markers of inflammation and oxidative stress in the lung receded after 3 days of recovery under normoxic conditions with NaCl infusion. In contrast, the PE did not resolve; on the contrary, it became even more severe. Contrary to our assumption, NE administration did not aggravate PE, but it also did not improve LV function. These unexpected results allow some conclusions to be drawn about the mechanisms involved in the development and progression of hypoxia-induced PE.

### 4.1. The Effects of Hemodynamic Changes Due to Hypoxia and NE Application

Hypoxic PE is considered to be a hydrostatic PE, the main cause of which is an increased pulmonary capillary pressure due to hypoxic pulmonary vasoconstriction (HPV) [9]. HPV is one of the earliest events in the response to hypoxia that occurs both in pulmonary resistance arterioles and venules [17] with vasoconstriction in venous vessels being particularly important for the increase in capillary pressure [38]. As HPV exhibits regional differences, this results in uneven distribution of pulmonary blood flow with an overperfusion of regions with weak HPV. Capillary pressure increases particularly in these hyperperfused areas, inducing filtration of fluid from the capillaries into the interstitium [9,39]. The elevated capillary pressure puts strain on the vessel walls, which is termed capillary wall stress. Excessively high pressure, which is not uncommon in HAPE, can lead to stress failure with ruptures of the blood–gas barrier (BGB) resulting in increased capillary leakage of fluid, proteins and cells [40,41].

The hemodynamic measurements of the present study confirmed previous results about the LVSP being significantly decreased under hypoxic conditions while RVSP even increased slightly [36,42]). NE infusion induced similar hemodynamic changes. The increased RVSP leads to an increase in pulmonary arterial pressure, which, in combination with HPV, also causes the pulmonary capillary pressure to rise. These effects of hypoxia (and similarly, of NE infusion) caused a significant imbalance between the systolic pressures of the LV and RV (see Figure 7), which can lead to blood congestion in the lungs and thus further increase pulmonary capillary pressure. Previous studies showed that such a mismatch between LVSP and RVSP in the early stage of edema formation (within the first 90 min of hypoxia exposure) aggravated the formation of PE [34]. However, pulmonary congestion under hypoxia or with NE infusion in the present study was not more severe than under normoxic conditions suggesting that at this stage, the hemodynamic situation and pulmonary congestion do not contribute significantly to edema formation.

One of the main compensatory mechanisms for increasing pulmonary capillary pressure and increasing fluid filtration into the pulmonary interstitium is fluid drainage into the pleural space, into the mediastinum and via the pulmonary lymphatic vessels [11,43,44]. In fact, we observed a marked increase in the amount of PF in the H-NaCl group, but this did not completely prevent the formation of edema.

Animal experiments have shown that an infusion of NE or of other adrenergic agonists can induce PE even under normoxic conditions [27,45], and this was confirmed in the present study. This pro-edematous effect of NE is well known in the human pathology. Neurogenic PE or PE as a complication of pheochromocytoma are examples of PE induced by adrenergic agonists [31,32,46,47]. The present findings in the NE-infused animals show even more clearly that the hemodynamic situation has only a minor influence on the degree of edema. In the N-NE group, the findings regarding hemodynamics and PF volume were similar; accordingly, the degree of edema was also in a similar range. In the H-NE group, however, the imbalance between LV and RV pressures was even greater than in all other groups. LVSP and SV were significantly lower than in the other groups, and LV edP reached approximately 1.3 to 1.8 times the values of the other groups. However, although these animals produced lower amounts of PF, congestion and edema in the lungs were even weaker than in the H-NaCl and N-NE groups. These results correspond to the characterization of HAPE as a non-cardiogenic pulmonary edema with elevated pulmonary capillary pressure and normal left atrial pressure [9]. Furthermore, they are consistent with previous observations in hypoxic rats that NE does not significantly aggravate PE and adrenergic blockers do not significantly reduce it [34,42].

The findings of the recovery groups also confirm the relatively minor influence of hemodynamic factors on the advanced stage of edema. After 3 days of recovery under normoxic conditions with NaCl infusion, the LVs function returned to approximately its initial level, which also reduced the imbalance between the LVSP and RVSP and blood congestion in the lungs. Nevertheless, the PEI increased during this recovery phase. But what could be the reasons for the edema persisting even after reoxygenation, despite pulmonary capillary pressures being presumably normalized?

### 4.2. The Role of Inflammation and Oxidative Stress

The PE was accompanied by inflammation, which was evident in the histological picture as well as in the expression of the proinflammatory cytokine TNFα. The histological signs of inflammation and the expression of TNFα were low under normoxic conditions both with NaCl and NE infusion. Hypoxia induced a significant increase in TNFα expression, and even more so with additional NE infusion. After 3 days of normoxic recovery, the TNFα level returned in all groups to the normoxic control level.

Hypoxia promotes the release of proinflammatory cytokines such as interleukin (IL)-1, IL-6 and TNFα [1,48,49]. Increased levels of these cytokines were found in the BALF of people at high altitude in their early stage of HAPE development. At admission, patients with HAPE presented TNFα concentrations in BALF of 12.6 ± 6.7 pg/mL on average. It should be noted, however, that five of seven patients only had concentrations ranging from 1 to 3 pg/mL. In the recovery phase, the TNFα concentrations decreased to <0.5 pg/mL [1]. Elevated cytokine concentrations were also observed in persons at high altitude who did not develop symptoms of HAPE [49]. Other studies demonstrated that the first HAPE symptoms preceded the occurrence of inflammation markers in BALF and that the BALF showed a non-inflammatory hemorrhagic picture with non-detectable TNFα concentrations in the first day of HAPE [17]. These results indicate that inflammation should not be considered the cause of HAPE [5]. Our results are completely consistent with this concept. This is impressively highlighted by the complete regression in TNFα expression to control levels after 3 days of recovery under normoxic conditions, while PE persisted after this period.

Hypoxia is also associated with increased production of reactive oxygen–nitrogen species (RONS). The reduced oxygen supply leads to disturbances in the mitochondrial electron transport chain [19]. Moreover, RONS-generating enzymes such as superoxide dismutase, NADPH oxidases or NO synthases are upregulated under hypoxia [20,21,50,51,52]. HIF-1 as one of the main regulators of response to hypoxia plays an important role. On the one hand, it may promote oxidative stress, but on the other hand, it can prevent excessive production of RONS [53,54]. Accumulation of RONS can induce oxidative damage to lipids, proteins and DNA. In particular, in addition to its cytotoxic effect, peroxynitrite directly alters proteins of the endothelial cytoskeleton, leading to dysfunction of the endothelial barrier of pulmonary arteries [23]. Peroxynitrite-induced damage to the vascular barrier can promote fluid accumulation in the lung and consequently, the formation of a permeability and hydrostatic PE, as demonstrated in a study on isolated rat lungs perfused with peroxynitrite [55].

RONS are also important mediators of inflammatory processes [25,52], which may promote and aggravate the formation of PE. A study on rats exposed to hypobaric hypoxia showed a significant increase in ROS generation as well as a significantly increased expression of NFκB and proinflammatory cytokines. These changes were associated with a marked increase in membrane peroxidation and vascular leakage [50]. Peroxynitrite also contributes to the proinflammatory effects of RONS [52]. Conversely, inflammation promotes the production of RONS and thus increases the damage to DNA and cell components. The potent proinflammatory cytokine TNFα has been demonstrated to induce the peroxynitrite-dependent increase in the permeability of pulmonary capillaries [56], indicating that inflammation and oxidative stress can maintain and aggravate edema formation. Although the animals in the recovery groups of the present study showed a clear regression of TNFα and NT levels, damage to the vessel wall has to be assumed, which was at least partly caused by inflammation and oxidative stress and was definitely exacerbated by these factors. The damage to the capillary wall requires a large number of repair and remodeling processes [57]. Some small endothelial or epithelial breaks can be closed within a few minutes when the basement membrane is still intact; however, complete closure of all disruptions is not achieved [58]. Complete repair is part of pulmonary remodeling that lasts days to weeks [59,60]. Hence, we would expect that the vascular leak would still persist after three days of recovery under normoxic conditions.

### 4.3. The Protein Content in PF and BALF

The significantly elevated protein concentrations in PF and BALF of the hypoxic or NE-infused rats clearly confirm an increased capillary permeability. A protein-rich PF with a specific gravity above 1.016 corresponding to a protein concentration above 30 g/L indicates an exudate, which typically results from increased vascular permeability [61]. In addition, a [P]_PF_/[P]_S_ ratio above 0.5 is one of Light’s classical criteria that distinguishes exudates from transudates [62]. Animals treated for 3 days with NaCl infusion and hypoxia presented with such elevated protein concentrations in PF. Hypoxia-induced PE is typically a hydrostatic edema, which may be aggravated by an increased vascular permeability due to capillary stress failure [40]. A protein-rich BALF confirming capillary stress failure and increased vascular leak has also been found in climbers to high altitude with HAPE [1,63]. The pulmonary blood–gas barrier (BGB) can withstand transmural pressures of up to approximately 24 mmHg. Above this value, first disruptions in the BGB may occur, but consistent breaks in the BGB have been found at pressure values around 40 mmHg [41]. In humans, pulmonary capillary pressures above 24 mmHg may be achieved during heavy exercise [64]. In athletes after heavy exercise in normoxia, BAL analysis revealed elevated levels in total protein concentration and red blood cells in the absence of inflammation markers, indicative of damage to the BGB with high-permeability edema [65,66]. Of note, red blood cell numbers and total protein concentrations increased even more when the same exercise protocol was performed under hypoxic conditions. The high concentration of red blood cells in BALF remained even after 26 h, indicating a persistent capillary leak [66]. While an elevated protein concentration in PF with a low protein level in BALF as found in the H-NaCl group suggests that disruptions mainly concerned the vascular side of the BGB so that fluid and protein filtration is confined to the interstitium, elevated protein levels in BALF as observed in the NE-infused rats signify that the disruptions pass through the entire BGB. This favors the assumption that NE aggravates capillary stress failure. Elevated capillary pressure is the primary cause of capillary wall stress and inflammation and oxidative stress further contributes to the damage to the BGB and vascular leakage. NE infusion or stress reinforced the imbalance between LV and RV systolic pressures, thus increasing capillary wall stress and promoting stress failure. Subjects prone to HAPE have been shown to present an exaggerated sympathetic nerve activity under hypoxic exposure, indicating that sympathetic overactivation might contribute to the development of HAPE by increasing pulmonary vasoconstriction [67]. In addition, previous studies on rats demonstrated that infusion of NE and other adrenergic agonists induced both edema and inflammation in the lungs even under normoxic conditions [28,45,68].

The protein concentrations in the recovery groups provide some information about the time course of the BGB repair. Previous studies demonstrated an initial mechanical repair in endothelial and epithelial breaks in the BGB within several minutes. The reversible breaks were mainly the smaller ones with intact basement membranes [58]. On the other hand, biological repair includes the proliferation of endothelial cells, angiogenesis, as well as vascular remodeling, all of which are processes requiring protein synthesis and therefore take several days [59,60]. In the H-NaCl+R group, the protein concentrations both in PF and in BALF were approximately at control level, indicating that the defects in the BGB were largely closed. In contrast, in the recovery groups after NE treatment (N-NE+R and H-NE+R), the protein concentrations decreased in BALF but increased in PF, suggesting largely closed defects on the alveolar side with persistent vascular leak.

The reduced protein concentrations in serum might be explained, on the one hand, as a result of the protein loss via the capillary leak and, on the other hand, by fluid retention due to reduced diuresis. While individuals climbing to high altitudes typically increase their diuresis [69,70], people with no increase in diuresis are prone to develop high-altitude diseases [71]. This might be a further contributing factor to the persistent PE in the recovery groups. Further studies should include measurements of fluid intake and output to assess more accurately the fluid volume changes in hypoxia and their significance for the development and persistence of HAPE.

### 4.4. Limitations to the Study

We cannot make any definite statements about the volume state of our experimental animals. As discussed above, it is well known that exposure to hypoxia increases diuresis [69,70], which may protect the lung capillaries from excessive pressure increases. Measurements of fluid intake and urine excretion would provide more detailed information on the volume state of the animals and its possible role in formation and resolution of hypoxic PE.

Another limitation of this study is that we only used female rats. This was performed to compare the results with previous studies [3,4,34,35,36,42]. Although blood pressure and heart rate were similar in male and female rats under hypoxic conditions [72], female rats are more tolerant to hypoxia and recover their respiration faster than males after hypoxic exposure [73]. Correspondingly, a recent review of HAPE in humans demonstrated that the incidence of HAPE in women averaged only about 14% of the incidence in men, with the percentage ranging from 2% to 37% in individual studies [74]. Hence, a comparison between rats of both sexes would be interesting and would probably show more severe pulmonary edema and injury in male animals than we found in the present study.

In the present study, we used a semiquantitative detection of TNFα in the lung tissue as a marker of inflammation. The inflammatory foci in lung tissue are distributed heterogeneously. Analyses using homogenized tissue such as ELISA or RT-PCR are not well suited to detect focal inflammation because regional differences are blurred. Therefore, immunohistochemical methods are more suitable to demonstrate inflammation markers in the hypoxic lungs, although they are only semi-quantitative. More quantitative methods such as ELISA or RT-PCR can be used to determine TNFα concentration in BALF. However, several studies in patients with HAPE revealed very low or even non-detectable TNFα concentrations in the BALF [1,17]. It should be noted that the relative amount of instilled fluid in these patients was much lower than in the rats of the present study (2 × 3 mL related to an average body weight of 243 g). Therefore, we would expect even lower TNFα concentrations in the BALF of our rats, possibly even below the detection threshold.

We decided not to count the cells in the BAL fluid because a previous study on rats exposed to hypoxia over 72 h revealed a normal, i.e., non-inflammatory distribution of cells without marked differences between treatment and control groups [4].

Our analyses give hints on pulmonary capillary stress failure. However, direct proof of disruptions in the alveolo-capillary barrier would require further experiments including vascular permeability assays, which have to be performed in vivo or in cell culture.

## 5. Conclusions

Our data show that rats exposed to hypoxia over three days develop PE, which is mainly confined to the interstitium. However, capillary stress failure may have occurred, as indicated by elevated protein levels in the PF or in the BALF. In addition, hypoxia induces inflammation and increased oxidative stress, which can also induce damage to the BGB. NE infusion can promote PE formation even under normoxic conditions, but the combined exposure of NE plus hypoxia did not aggravate the severity of PE despite a mild further increase in TNFα and NT as markers of inflammation and oxidative stress. Similarly, NE plus hypoxia enhanced the LV depression and the imbalance between LV and RV pressures without increasing the severity of PE. These findings are in full line with the widely accepted definition of HAPE as a hydrostatic but non-cardiogenic PE, which may result in a permeability edema due to capillary stress failure [9,75]. Although edema is accompanied by inflammation, this develops in parallel with the edema and therefore is considered not to play a causal role in the development of the edema [5]. This is further emphasized by the results of the recovery animals. Despite a complete regression of the markers of inflammation and oxidative stress, PE persists even after three days of recovery in normoxia and with NaCl infusion. It is reasonable to assume that not all breaks in the BGB have been repaired and closed during this short period of time so that fluid and proteins still can pass through the capillary wall and maintain the PE.

## Figures and Tables

**Table 1:** Hemodynamic data.

	-------------- Treatment Groups --------------				-------------- Recovery Groups --------------			
	N-NaCl	H-NaCl	N-NE	H-NE	N-NaCl+R	H-NaCl+R	N-NE+R	H-NE+R
LVSP [mmHg]	123.3 ± 3.4 #	103.5 ± 3.8 *	106.7 ± 4.1 *	92.1 ± 3.4 *#+	102.0 ± 5.5 $°	123.8 ± 3.8 §°	131.1 ± 3.8 #§°	120.9 ± 3.3 #§°
RVSP [mmHg]	30.9 ± 0.9	34.3 ± 1.5	34.5 ± 2.4	38.5 ± 1.5	30.1 ± 2.7	33.9 ± 1.2	33.0 ± 1.8	33.7 ± 1.1
MAP [mmHg]	109.6 ± 3.5 #	91.4 ± 3.6 *	94.7 ± 4.0 *	77.9 ± 3.0 *#+	91.4 ± 5.6 $°	111.9 ± 3.2 §°	117.2 ± 3.5 #§°	108.0 ± 2.9 #§°
SV [µL]	215.3 ± 7.2	185.6 ± 11.6	161.6 ± 11.5 *	131.2 ± 20.0 *#+	215.5 ± 13.9	214.2 ± 18.5	192.3 ± 23.5 §$	220.1 ± 7.9 °
HR [min^−1^]	441.8 ± 8.1 #	410.4 ± 8.3 *	451.0 ± 10.5	436.2 ± 8.6	431.3 ± 13.0	457.0 ± 12.0 °	423.4 ± 7.0 $°	415.8 ± 9.4 $
CI [mL min^−1^ kg^−1^]	397.7 ± 16.7	334.9 ± 18.5	299.9 ± 19.0	223.8 ± 32.5 *	392.4 ± 24.1	388.5 ± 29.1	316.5 ± 29.3	357.6 ± 14.2
LV edP [mmHg]	5.93 ± 0.54	6.38 ± 0.35	4.58 ± 0.38	8.08 ± 0.43 +	5.24 ± 1.74	5.20 ± 0.59	5.58 ± 0.39	5.68 ± 0.86
TPR [mmHg min kg mL^−1^]	0.29 ± 0.01	0.27 ± 0.02	0.33 ± 0.02	0.43 ± 0.08	0.24 ± 0.02	0.30 ± 0.02	0.41 ± 0.06 §	0.31 ± 0.01

LVSP, left ventricular systolic pressure; RVSP, right ventricular systolic pressure; MAP, mean aortic pressure; SV, stroke volume; HR, heart rate; CI, cardiac index; LV edP, left ventricular enddiastolic pressure; TPR, total peripheral resistance. Significance symbols: * significant vs. N-NaCl, # significant vs. H-NaCl, + H-NE significant vs. N-NE; recovery groups: § significant vs. N-NaCl+R, $ significant vs. H-NaCl+R, ° significant vs. corresponding treatment group.

## Data Availability

The raw data supporting the conclusions of this article will be made available by the authors on request.
